# General Survey of England and Wales

**Published:** 1920-05-29

**Authors:** Napier Burnett


### THE HOSPITAL SITUATION.

### General Survey of England and Wales.

By Sir NAPIER BURNETT, K.B.E., M.D.

Throughout the fifty-five counties (England and Wales) 550 hospitals have been dealt with up to date, approximately 78 per cent. of the voluntary civil hospitals, omitting those dealing exclusively with tuberculosis. The Voluntary Hospitals in London are not included in the present survey, as it was felt that for many years the London hospitals have enjoyed the benefit of the great collecting organisations, such as King Edward's Hospital Fund, the Hospital Sunday Fund, the Hospital Saturday Fund, and the League of Mercy. The provincial hospitals have had no such central funds, and this fact suggested priority of assistance being given to these institutions.

### Tabular Statement of the Survey.

A.—Volume of Work Done.

Of the 550 hospitals reviewed :

507 hospitals have 29,821 available beds.
498 ,, treated 350,459 in-patients during the year.
376 ,, treated 1,600,869 out-patients during the year.
296 ,, with 23,621 beds had a daily average occupation of 18,705.36 beds = 79 per cent.
374 ,, show 219,196 surgical operations during the year.
68 ,, give figures showing the nature of cases treated, namely :—

*Medical Cases* 7,034 = 21 per cent. of total.
*Surgical Cases* 26,466 = 79 per cent. of total.

B.—Financial Position.

543 hospitals show ordinary income £2,835,269.
543 hospitals show ordinary expenditure £3,310,896.
Excess of expenditure over income £475,627.

*Some details of ordinary income* :

316 hospitals show as workmen's contributions, direct or through Saturday Fund—£451,426 = 15.92 per cent. of ordinary income of 543 hospitals.
464 ,, show as patients' contributions—£214,570 = 7.56 per cent. of ordinary income of 543 hospitals.
248 ,, show as public services (*e.g.*, War Office, Pensions Ministry, Borough or County Councils)—£517,890 = 18.91 per cent. of ordinary income of 543 hospitals.
512 ,, show as interest from investments—£423,044 = 14.92 per cent. of ordinary income of 543 hospitals.

The total from these four sources—£1,606,930 = 56.67 per cent. of ordinary income of 543 hospitals.

Of these 543 hospital accounts analysed :

449 hospitals show invested capital as £9,585,865
Yielding annual interest 340,529
66 ,, show £33,706 as annual interest, but show no capital.
28 ,, show no capital or interest.

### Depreciation of Invested Funds.

9 Hospitals show invested capital as ... £306,956
Market value at end of 1919 being ... £248,199

**Depreciation in Value** ... ... £58,757

This last item is shown merely in answer to a criticism that the hospitals in their present financial difficulty should realise some of their capital. Some few hospitals have actually been obliged to take this course, but only very few. The figures indicate that the hospitals cannot afford to unload their stock in the present state of the market.

In order to show the situation more clearly, I have divided the hospitals into the following groups, namely :—

| No. of Hospitals | No. of Beds | Average No. of Beds to each Hospital | Total Ordinary Income | Total Ordinary Expenditure | Excess of Expenditure over Income |
|---|---|---|---|---|---|
| *A.—Large General Hospitals of 100 Beds or over.* | | | | | |
| 82 | 15,958 | 194·60 | £1,615,291 | £1,970,976 | £355,685 |
| *B.—Medium-sized Hospitals of from 30 to 100 Beds.* | | | | | |
| 87 | 4,724 | 54·29 | £436,331 | £476,049 | £39,718 |
| *C.—Small or Cottage Hospitals of under 30 Beds.* | | | | | Excess of Income |
| 240 | 3,335 | 13·97 | £289,489 | £276,855 | £12,634 |
| *D.—Special Hospitals.* | | | | | Excess of Expenditure |
| 98 | 5,784 | 59·02 | £458,309 | £540,227 | £81,918 |

| Dispensaries | New Out-Patients | Total Ordinary Income | Total Ordinary Expenditure | Excess of Income over Expenditure |
|---|---|---|---|---|
| *E.—Out-Patient Clinics.* | | | | |
| 43 | 165,159 | £51,838 | £50,598 | £1,240 |

### Comments on the Survey Figures.

So much has been written of late *re* hospitals, especially in the form of general statements, that the figures brought out in the Survey may be regarded as dealing with "the facts" of the situation. They are issued as a first instalment dealing with some 550 hospitals—or approximately 78 per cent. of all provincial hospitals—a sufficiently broad basis to draw conclusions from. Further details will be published at a later date showing more particularly the voluntary hospitals, with their figures in each individual county in reference to population, etc.

Two additional figures, not shown in the above statement, were taken out, namely (*a*) the average length of stay in hospital of each in-patient; (*b*) the average annual cost per occupied bed.

The methods employed by some of the hospitals in ascertaining these data varied so much that I had no option but to discard the results. For example, some hospitals included their "tonsil and adenoid" cases—usually in hospital only twenty-four hours—while other hospitals excluded these, in arriving at their "average length of stay" figure. Again, in regard to "cost per occupied bed" I found that some hospitals included the cost of out-patients, while others deducted such expenditure, so that I concluded the figures, for purposes of comparison, were unreliable.

### The Year's Work.

The volume of work done at the 550 hospitals reviewed shows that approximately two million patients received treatment during the year. The figure shown for out-

**The Hospital Situation**—*(continued)*.

patients indicates individual cases, and is not merely a record of "attendances."

During the war, owing to depleted clerical staff, many of the hospitals allowed certain details to lapse from their records. Hence it is that only 296 of the 550 hospitals give the "average daily occupation of beds"; but where it has been given it reveals the high occupation of 79 per cent. This is undoubtedly high when taken for the whole year, during part of which, as a rule, some ward or wards are closed for cleaning or disinfection purposes. A further testimony of the enormous volume of work done in these hospitals is seen in the figure of 219,196 surgical operations during the year. These included both major and minor operations where an anæsthetic has been employed, but do not include dental operations.

One other interesting figure is shown under the ratio of medical to surgical cases treated. It is true that only some 68 hospitals give these figures, but they are sufficiently numerous as to indicate a present-day tendency towards catering for surgical work. I suggest that these figures do not indicate that medical diseases are such a disappearing quantity, but rather that people no longer shun the hospital in dread of an operation. Although much remains to be done in the direction of the standardisation of surgical work as is now being attempted in some of the American hospitals, yet it would appear that the general public are gaining increased confidence in the results of modern surgery. On the other hand, the gradual exclusion of medical patients is a matter of some importance. Not infrequently one has been told by doctors of their difficulties in getting patients suffering from such complaints as kidney or heart disease, etc., into hospital. It may be that such medical cases may be better treated in the quieter atmosphere of the convalescent home, rather than amidst the turmoil and bustle of a general hospital in the centre of a large town or city.

### Finances and Needs.

Turning to the financial position, it will be seen that the excess of expenditure over income for the hospitals under review amounts to £475,627. Approximately 75 per cent. of this deficit belongs to the large general hospitals of 100 beds or over, of which there are 82 in number. It would appear from these figures that big hospitals situate in the populous industrial centres are supplying a much larger service than is recognised by the local communities. The classification of the hospitals reviewed into groups according to their number of beds, brings out more clearly the financial position. The difficulties at present being experienced by the voluntary hospitals may be grouped under three headings, namely—(*a*) Need of increased financial support; (*b*) need of increased bed accommodation; (*c*) need of increased supply of nurses.

Appended hereto is a suggested scheme for assisting the voluntary civil hospitals.

### The Joint Council's Peace Programme.

1.—Hospital Survey.

The survey will consist of (*a*) Data showing the volume of work done during the year 1919 in each individual hospital. (*b*) The financial statement of each hospital, showing ordinary income and expenditure, invested funds, annual cost per bed, etc. (*c*) Showing by maps the hospital situation in each county in ratio of beds to population.

2.—Public Appeal for Funds.

The sum of £1,000,000 per annum is aimed at—to be distributed in the form of grants, supplementary to the individual efforts of the hospitals themselves and the other welfare agencies.

The money, it is hoped, will be raised from the following sources, *viz.*: (*a*) Public appeal through the Press, etc., for individual subscriptions. (*b*) Public appeal and personal canvass of business firms, pointing out to commercial organisations the point of view that, by setting aside each year a certain sum of money for distribution amongst charities, may be regarded as a reasonable tax on industry, and that by handing such sums to the Red Cross organisation for distribution they may be assured that the money will be distributed where it is most needed, or if ear-marked for certain districts it will be so distributed, and thus relieve the business man of the uncertainty of knowing who is in most need of his support. (*c*) Public appeal through the great national collecting agency, known during the war as "Our Day" organisation.

The allocation of grants to hospitals will be made by a Grants Committee, on the recommendation of the Director of Hospital Services, after investigation of such matters as: (*a*) The efficiency of the hospital administration. (*b*) How far the hospital is meeting the needs of the locality. (*c*) As to its buying on scientific lines, prices paid, etc. (*d*) As to adequate payment of its nursing staff, always provided that the standard of training is of the highest, etc., etc.

3.—Co-operative Buying of Hospital Commodities.

In some instances hospitals are buying in competition with each other in the same locality, and in one area where the figures were examined there was found a wide range of difference in the prices paid for the same commodity. Hospital expenditure might be appreciably curtailed by some system of co-operative buying.

The British Red Cross Society has large storage accommodation in London where non-perishable articles could be stored in bulk, and some of the advantages which the hospitals could derive under this aspect of the scheme are: (*a*) Buying in bulk. (*b*) Buying on analysis. (*c*) Obtaining the best information as to markets, etc.

In asking the public for money we must seek to assure the public that their money is being spent to the best advantage.

4.—Co-ordinating the Hospitals into Groups in Counties or other Areas.

In every county or district there is the large central hospital which may be called the "key" hospital, and it is hoped to arrange with the smaller county and cottage hospitals to become co-ordinated with the key hospital by having a working arrangement as to: (*a*) Transport of patients. (*b*) The central hospital to receive severe cases from the smaller hospitals and return of slighter cases to the cottage hospitals and convalescent homes. (*c*) All pathological and bacteriological work of each group of hospitals to be arranged for at the central hospital. No hospital would receive a grant from the Joint Council unless some such arrangements for the investigation of disease existed, etc., etc.

5.—Circulation of Statistical and other Hospital Information from the Central Bureau.

Many hospitals possess some individual point of excellence, which at present is unknown to other hospitals. These special points would be collected and circulated for the good of all.

6.—Convalescent Hospitals.

To give every encouragement for the establishment of convalescent hospitals in the country for the dual purpose of: (*a*) *Pre-hospital.*—Preventing many thousands of

**The Hospital Situation—(*continued*).**

people from graduating into hospital patients by granting them the benefit of fresh air and rest. (*b*) *Post-hospital.*—To relieve the pressure on the present overtaxed hospital accommodation, and allow other serious cases to be attended to, and at the same time to accelerate their convalescence and speedy restoration to their employment.

7.—Meetings to be arranged in each county with the object of disseminating knowledge and stimulating interest in all matters pertaining to public health.

By means of some such scheme as that outlined above it is hoped to assist in the development of a national co-ordinated hospital system for the cure and prevention of disease.

